# One-stage surgery by a halo-vest to treat simultaneous double spine fractures–dislocation in a patient with ankylosing spondylitis: case report and review of literature

**DOI:** 10.3389/fsurg.2024.1383550

**Published:** 2024-10-29

**Authors:** Liang Wang, Youcai Qiu, Can Wang, Tielong Liu, Xuhua Lu

**Affiliations:** ^1^ School of Health Science and Engineering, University of Shanghai for Science and Technology, Shanghai, China; ^2^ Department of Orthopaedics, Second Affiliated Hospital of Naval Medical University, Shanghai, China; ^3^ Department of Orthopaedics, Affiliated Hospital of North Sichuan Medical College, Sichuan, China

**Keywords:** ankylosing spondylitis (AS), halo-vest immobilization, cervical spine trauma, simultaneous double spine fractures–dislocation, surgery

## Abstract

**Study Design:**

A case report.

**Background:**

In this study, we report the outcome of one-stage surgery using a halo-vest to treat simultaneous double spine fractures–dislocation in a patient with ankylosing spondylitis (AS).

**Case presentation:**

We report a case of a 57-year-old male patient with AS who sustained simultaneous double spine fractures due to a traffic accident. We performed an open approach after successful closed reduction using a halo-vest. At the 1-year follow-up, we finally achieved bone union after postoperative. At the 3-year follow-up visit, he reported a significant resolution of both cervical and back pain and had returned to his routine daily activities.

**Conclusion:**

This is the first report of using a halo-vest to treat simultaneous double spine fractures–dislocation in a patient with AS. The authors included five research studies that situate this case study in the existing literature and highlight a gap in current knowledge. Based on our experience with this case and a review of the literature, one-stage surgery by a halo-vest is an effective option for the treatment of simultaneous double spine fractures–dislocation in patients with AS.

## 1 Introduction

Ankylosing spondylitis (AS) is a common chronic inflammatory disorder involving axial joints, peripheral joints, and even extra-articular organs (1). Compared with the normal population, patients with AS are approximately 3–5 times more likely to experience cervical spine fractures (2). The fractures are usually associated with low-energy trauma (3). Fractures often involve three columns, and the spine becomes very unstable. AS-related fractures are often more serious than fractures in the healthy population and frequently recognized with delay, which increases the risk of secondary neurologic injury due to high instability and potential displacement (4). Fractures often necessitate surgical intervention, but surgical planning and execution are oftentimes challenging due to the instability of the fracture. To our knowledge, simultaneous double spine fractures–dislocation in patients with AS have been rarely reported in the literature (5–9). The treatment strategies for simultaneous double spine fractures–dislocation in patients with AS have to be different from patients with non-rigid spines. We report a case of a patient with AS who was treated with a halo-vest for simultaneous double spine fractures–dislocation. This study aimed to investigate the efficacy of one-stage surgery by a halo-vest for treating AS with simultaneous double spine fractures–dislocation.

## 2 Case report

**Figure 1:**
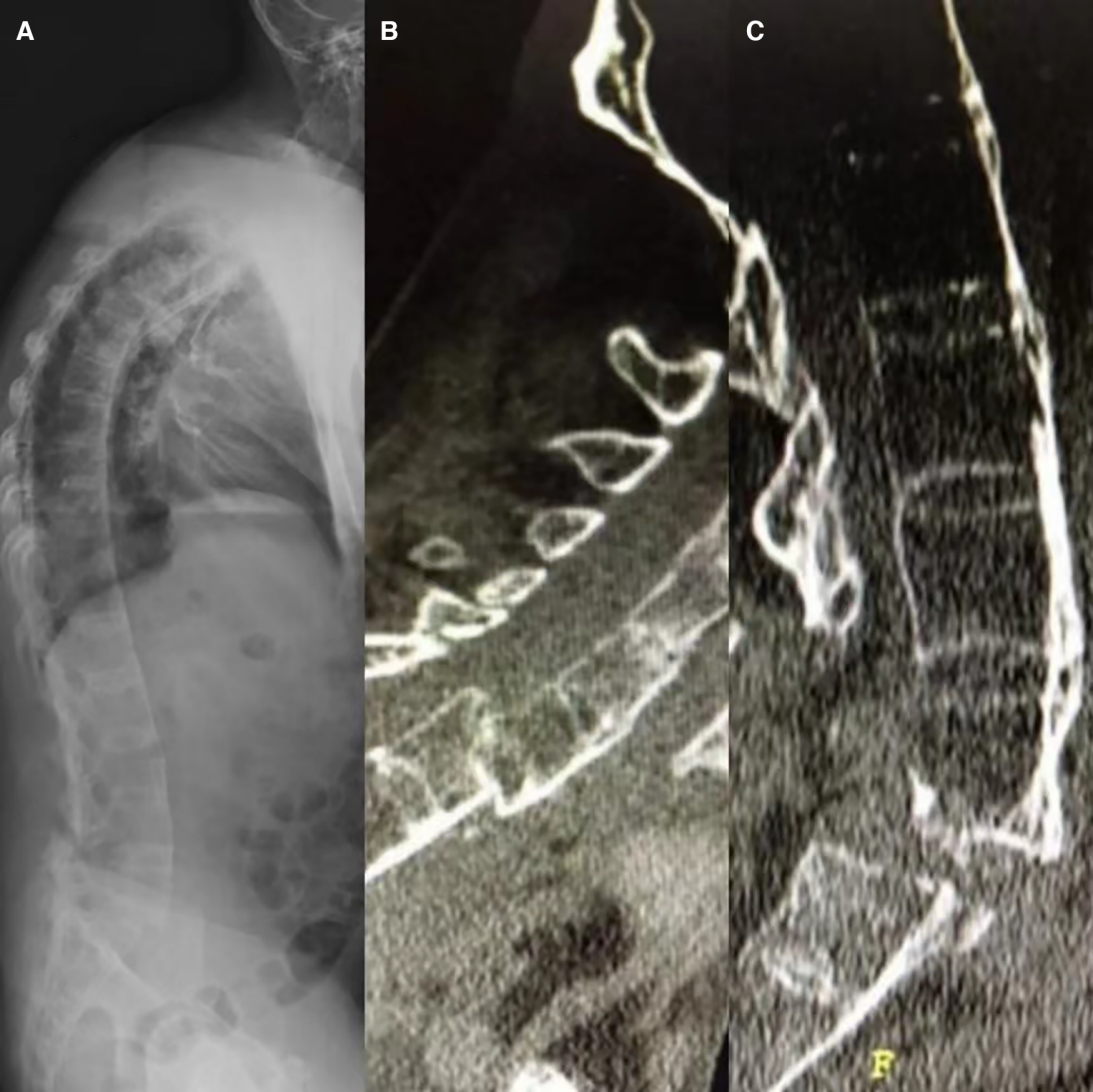
A 57-year-old male with ankylosing spondylitis presented with a lumbar fracture (L4–5). Plain radiographs of the full spine were unremarkable in detecting a cervical spine fracture in this patient **(A)**. A sagittal CT scan also revealed a displaced and unstable fracture–dislocation of C6/7 **(B)** and L5/S1 **(C)**.

A 57-year-old male patient with AS developed paralysis after a traffic accident. On admission, he complained of intractable neck and back pain and also reported numbness and weakness in the limbs. Physical examination revealed an incomplete paraplegia. His American Spinal Injury Association (ASIA) classification was C. The right upper limb had a muscle strength grade of 0, and the left upper limb had a muscle strength grade of 1. Lower extremity activity was not bad, with a muscle strength grade of 2. There was no muscular atrophy or hypertrophy and no superficial sensation. Corneal reflex and light reflex existed. Abdominal wall reflex, testicular reflex, and anal reflex were not elicited, and knee–tendon reflex was hyperactive. The Hoffman signs of both upper limbs were negative, and bilateral Babinski signs were negative in the lower extremities. A lateral plain radiograph revealed a fracture–dislocation of L5/S1, with involvement of all three columns, subluxation [a spinal fracture through the L4–L5 intervertebral disc (arrow) up to the posterior vertebral elements completely dissecting the spine]. Plain radiographs of the cervical spine were unremarkable in detecting a cervical spine fracture in a patient with AS. A computed tomography (CT) scan of the spine was performed demonstrating a displaced and unstable fracture–dislocation of C6/7 and L5/S1 (Figure 1). The majority of the fractures have long, oblique lines, involving two or more vertebrae, and are accompanied by bone defects in the anterior centrum and one to two spinous process fractures above the rear fracture lines. Both fractures were deemed to be unstable. His erythrocyte sedimentation rate (ESR), C-reactive protein (CRP), HLA-B27 levels, and white blood cell (WBC) and neutrophil counts were normal. The diagnosis was as follows: C6 fracture, dislocation, and spinal cord injury (SCI); L5/S1, fracture, dislocation; AS.

**Figure 2:**
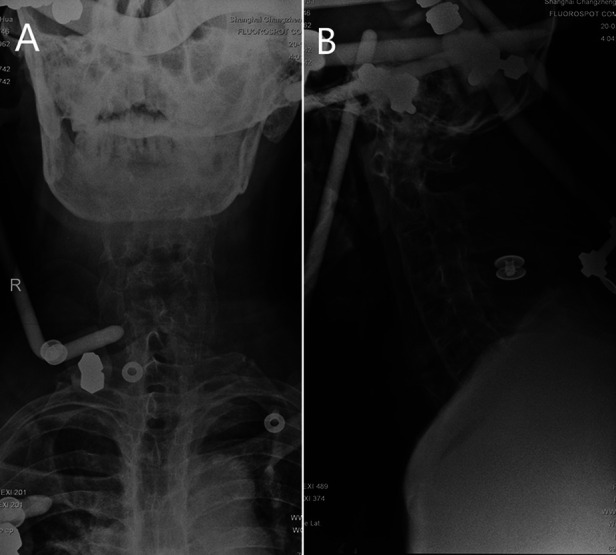
Plain radiographs of the cervical spine revealed closed fracture reduction was performed with the use of a halo-vest **(A,B)**.

Cervical external fixation with a halo-vest was performed the next day after the injury, and the reduction was successful (Figure 2). After careful analysis, the decision was made to fix the fracture and correct cervical fracture–dislocation from the combined anterior and posterior approach and lumbar fracture–dislocation from the posterior approach. The patient underwent intraoperative neurophysiologic monitoring including sensory and motor-evoked potentials during surgery. To restore cervical stability, a combined posterior and anterior fixation approach was performed on the patient who underwent an anterior autologous iliac bone interbody fusion because of a sizeable anterior gap. Posterior fixation was strong and stable with few implant failures, and the fixed region was sufficient with two segments above and two below the fracture segment. Cervical radiographs revealed a good reduction of the fracture. After cervical surgery, to treat the lumbar fracture, lumbar fractures were treated operatively approach with posterior segmental instrumentation and fusion.

The whole procedure, from the positioning of the patient to wound closure, took 6 h 35 min.

**Figure 3:**
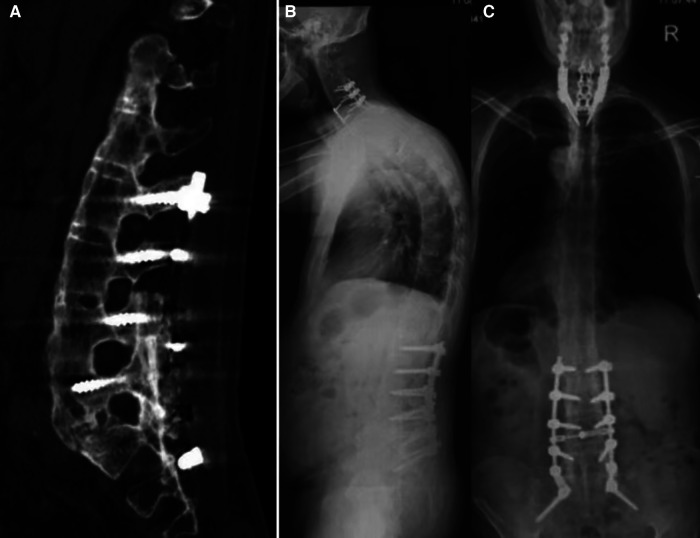
CT **(A)** and x-rays **(B,C)** at the most recent follow-up of the spine demonstrating healing of the fracture.

The operation was very successful. A postoperative double-check CT scan revealed that the internal fixation device was firm and reliable, and the stability of the spine recovered well. Postoperatively, the patient was kept in a halo-vest for 1 month. His neurological examination showed improvement, and his hospital course was uncomplicated. At the 1-year follow-up, no further complications occurred. The patient was able to walk around without back pain and intermittent radiating pain after postoperative one year. At the 3-year follow-up visit, he reported a significant resolution of both cervical and back pain and had returned to his routine daily activities. The patient’s ASIA classification improved from C to D. An x-ray and CT during his last visit were obtained demonstrating healing of the fracture (Figure 3).

## 3 Discussion

Vertebral spinal fractures are common in patients with AS and may lead to severe neurologic deterioration and even mortality (10). In patients with AS, the most common spinal fracture occurs in the cervical spine (53%), with an associated spinal cord injury occurring 27.5% of the time (11). Ectopic bone formation and reduced bone quality result in a further increased risk of fractures (12). Up to 14% of AS patients will experience a clinically manifest vertebral fracture during their lifetime. For patients with a spinal cord injury, there is a significantly increased risk of complications and a prolonged hospital stay. Mortality and morbidity are high after spinal injury in patients with AS, with death rates being as high as 35% in cervical fractures (13). Multiple fractures can be easily missed in AS patients and with devastating consequences (14). Throughout the studies abstracted and reviewed, there were a persistent number of injuries that had been missed or where patients experienced a delay in diagnosis.

In AS patients, spinal fractures are often missed as they are typically caused by low-energy injuries, with negative plain radiograph findings (15). X-rays may be hard to interpret, and fractures are missed in as many as 59.4% of patients, particularly in the lower cervical spine (16). A delay in diagnosis was found to occur in approximately 20% of patients with AS (17). An et al. showed that plain radiographs of the AS spine may fail to reveal a fracture due to the ossified ligaments and distorted anatomy; thus, an initial CT or MRI of the whole spine is recommended (18). Schiefer et al. highlighted the need for CT scans to image the spine and found 7% of patients had non-contiguous fractures; therefore, they advocated a low threshold for imaging the whole spine.

Because of the extensive ankylosis formation, fractures often extend through the disc space and often involve both anterior and posterior elements thus making them highly unstable (16). The treatment of this group of simultaneous double spine fractures–dislocation in patients with AS is complex. Due to the high risk of fracture and dislocation in simultaneous double spine fractures–dislocation in patients with AS, secondary neurological deterioration and progressive deformity may occur, resulting in a poor clinical prognosis. The halo-vest is a simple and safe immobilization tool that is widely used for the treatment of spine fractures in patients with AS. It can be fixed in three dimensions and can also facilitate complete three-dimensional adjustment of the cervical spine; that is, anterior flexion, lateral flexion, and rotational movement can all be controlled by adjusting the stent (19). The effect of a preoperative halo-vest for the reduction of spine fractures–dislocation in patients with AS has also been reported. Yang et al. used the halo traction method for AS patients with spine fractures–dislocation, and reduction was achieved by distracting the halo-vest in stages (20). In our current study, we applied preoperative progressive halo-vest traction to reduce cervical spine fracture–dislocation in patients with AS. The use of a halo-vest is associated with a high rate of successful fracture reduction. However, during the turning maneuvers, fracture displacement can occur even with precise techniques. Therefore, the application of a halo-vest makes positioning and induction of general anesthesia safer anf more convenient.

It is well known that patients with AS with displaced spine injuries have a high rate of spinal cord injury (21). Therefore, early aggressive surgeries with fixation are recommended. Although various sophisticated surgical techniques have been developed to explore the treatment of simultaneous double spine fractures–dislocation in AS patients, the management of these patients with simultaneous double spine fractures–dislocation may still be complicated by factors including high risk of limited spinal motion, osteoporosis, potential clinical complications, or neurological injury. In this report, we presented a case of an AS patient who suffered simultaneous double spine fractures–dislocation. Simultaneous double spine fractures–dislocation in patients with AS may be more common than those reported in the literature. What makes our case unique are the multiple considerations regarding closed reduction, positioning, and fixation options with a halo-vest. The feared complications after fracture– dislocation in patients with AS and the development of neurological deficits are well known; therefore, reliable external fixation is very important before and during surgery.

For unstable AS spine injuries, conservative management is not recommended. Treatment is controversial: some surgeons have reported a higher risk of complications after surgery, while others prefer using surgical fixation to avoid the risks related to conservative treatment (22). Many surgeons do not routinely employ percutaneous fixation techniques to treat patients with ankylosing spondylitis fractures, preferring to stabilize and fuse via an open approach. A long posterior stabilizing surgery is typically recommended (23, 24). Our decision to decompress and fix the cervical vertebra first was based on the progressive neurologic deterioration and the morbidity associated with unstable long bone fractures. Therefore, the combined anterior–posterior approach is the most appropriate choice for restoring and maintaining cervical stability and long posterior segmental instrumentation and fusion for lumbar stability.

Increased risk of spinal cord injury due to instability of the fracture necessitates careful consideration of transfers and operative positioning (10). In this study, we adopted a one-stage surgery using a halo-vest to treat complex, severe AS complicated by simultaneous double spine fractures–dislocation, achieving favorable outcomes. In fact, our literature review uncovered only five reports of AS and simultaneous double spine fractures–dislocation. Simultaneous double spine fractures in patients suffering from AS have been shown in multiple research studies, as seen in Table 1. Ushijima et al. presented a case of non-contiguous fractures in the cervicothoracic and thoracolumbar junction zones resulting from multiple injuries sustained in a traffic accident. The fractures were treated with hybrid techniques for posterior instrumentation with an open approach using a computed tomography (CT)-based navigation system and percutaneous pedicle-screwing method (5). The case reported by Samartzis et al. had simultaneous fractures at C6–C7 and L2–L3 in the second episode, and the patient died following the fractures (6). A previous study reported a patient with AS who sustained simultaneous double spine fractures and died (7). Krunal et al. presented a case involving simultaneous navigated cervicothoracic and thoracolumbar fixation of simultaneous double spine fractures–dislocation in a patient with AS (9). Arjun et al. also presented a novel approach combining open and percutaneous surgical techniques for treating multiple non-contiguous spinal fractures in a patient with AS (8). Their case was treated by fusion with instrumentation and posterior segmental instrumentation and fusion. In contrast, our case is therefore unique in that closed fracture reduction was performed with the use of a halo-vest to prevent further neurologic demise and facilitate postoperative recovery. Further research needs to be performed to determine the safety of using a halo-vest for treating simultaneous double spine fractures–dislocation in patients with AS.

**Table 1:** Case review of simultaneous spine fractures in patients with ankylosing spondylitis (AS).

Authors, year	Sex	Age	Location	Associated conditions Traumatic mechanism	Clinical presentation	Treatment
Ushijima et al., 2018 (5)	M	46	C6–C7, T12-L1	Traffic accident	No neurological deficit	Posterior instrumentation
Samartzis et al., 2005 (6)	M	81	C6–7, L2–3	Fall	ASIA A spinal cord injury	posterior segmental instrumentation and fusion
Yagi et al., 2015 (7)	M	82	C6–7, L1	Low-energy fall	ASIA A spinal cord injury	Death
Sebastian et al., 2015 (8)	M	77			Open fusion and percutaneous instrumentation techniques	Open fusion and percutaneous instrumentation techniques
Patel et al., 2018 (9)	M	49	Three-column fractures at C6 and T12	Fall	Intractable neck and back pain, neurologically intact	Cervicothoracic and thoracolumbar fixations simultaneously

## 4 Conclusions

Simultaneous double spine fractures in patients with AS are often missed. When patients with AS are suspected of multiple fractures and when the x-ray is negative, 3D-CT should be performed actively. Care should be taken in identifying spine fractures of the entire spine in patients with AS. This report is first to describe the use of a halo-vest for the treatment of simultaneous double spine fractures–dislocation in patients with AS. The authors included five research studies that situate this case study in the existing literature and highlight a gap in current knowledge. Based on our experience with this case and a review of the literature, one-stage surgery using a halo-vest is an effective option for simultaneous double spine fractures–dislocation in patients with AS.

## Data Availability

The original contributions presented in the study are included in the article/Supplementary Material, further inquiries can be directed to the corresponding author.
